# Sex and Age Differences in the Impact of Metabolic Syndrome on Heart Failure Development

**DOI:** 10.3390/metabo14120653

**Published:** 2024-11-25

**Authors:** Tae-Eun Kim, Do Young Kim, Hyeongsu Kim, Sung Hea Kim

**Affiliations:** 1 Department of Clinical Pharmacology, Konkuk University Medical Center, Seoul 05030, Republic of Korea; 2 Division of Cardiology, Department of Internal Medicine, Ajou University Hospital, Ajou School of Medicine, Suwon 16499, Republic of Korea; 3 Department of Preventive Medicine, School of Medicine, Konkuk University, Seoul 05030, Republic of Korea; 4 Division of Cardiology, Department of Internal Medicine, Konkuk University Medical Center, Konkuk University School of Medicine, Seoul 05030, Republic of Korea

**Keywords:** metabolic syndrome, heart failure, sex difference, young age

## Abstract

Metabolic syndrome (MetS), a cluster of metabolic dysregulations, is recognized as a significant risk factor for the development of heart failure (HF). The pathophysiological mechanisms linking MetS to HF are complex and multifaceted, with the components of MetS contributing to cardiac deterioration through impaired myocardial energy metabolism, increased inflammation, and endothelial dysfunction. Numerous clinical studies have confirmed the relationship between MetS and HF. Multiple studies have demonstrated that the impact of MetS on HF varies by sex and age. Metabolic disorders, including MetS, have a greater impact on HF incidence in younger adults compared to the elderly population and in women compared to men. Although the reasons for these differences are not yet fully understood, recognizing the sex- and age-related variations is crucial for developing targeted strategies to prevent HF in individuals with MetS. Future research should continue to investigate the underlying mechanisms behind these variations and identify optimal management approaches that account for both sex and age in reducing HF risk.

## 1. Introduction

Metabolic syndrome (MetS) involves a cluster of metabolic dysregulations comprising central obesity, insulin resistance, atherogenic dyslipidemia, and hypertension. The development of MetS is driven by a myriad of multiple genetic and acquired factors that contribute to insulin resistance and chronic low-grade inflammation [1]. Without proper management, MetS significantly elevates the risk of cardiovascular disease [2,3] and premature death [3,4]. A recent report analyzing US National Health and Nutrition Examination Survey data during 2011–2018 showed a steady increase in the prevalence of MetS since 2011, with the prevalence of MetS being 37.6% in 2011–2012 and 41.8% in 2017–2018 [5]. 

Heart failure (HF) is a clinical syndrome caused by a structural and/or functional abnormality of the heart, presenting with symptoms and signs associated with organ hypoperfusion and pulmonary or systemic congestion [6]. Affecting over 64 million people worldwide, HF is associated with significant morbidity and mortality, a poor quality of life, and a substantial strain on healthcare systems [7]. HF represents a significant healthcare challenge, affecting approximately 3% of the general population and serving as the leading cause of mortality, hospitalizations, and healthcare costs in individuals over 65 [8]. Population studies estimate the average lifetime cost following an HF diagnosis to be USD 109,541 per person, with hospitalizations [9] accounting for more than 75% of this expenditure, particularly in the final months of life [10].

Multiple independent groups have identified MetS as a significant risk factor for the development of HF. The pathophysiological mechanisms linking MetS and HF are complex and multifaceted, involving both direct and indirect effects on the cardiovascular system. The components of MetS, central obesity, hypertension, dyslipidemia, as well as insulin resistance, a hallmark of MetS, contribute to left ventricular dysfunction through various mechanisms such as impaired myocardial energy metabolism, increased inflammation, and endothelial dysfunction, leading to cardiac deterioration in individuals with MetS [11,12,13,14]. The pathophysiological link between MetS and HF is confirmed by many clinical studies. Miura et al. [15] observed that the prevalence of MetS is twice as high among individuals with HF compared to the general population. Similarly, Li et al. [16] found that MetS was linked to nearly a two-fold increase in the likelihood of self-reported HF in a population-based cross-sectional study. Moreover, some studies have highlighted the connection between MetS and HF prognosis [17,18,19].

Based on multiple reported findings, it appears that the impact of MetS on HF is different according to sex and age. Understanding sex- and age-related variations is crucial for developing targeted strategies to prevent HF in individuals with MetS and for improving patient outcomes through tailored management approaches. However, there are only fragmented reports on these sex- and age-related differences, and an in-depth understanding to explain these phenomena has not yet been established. Therefore, this paper aims to explore the mechanisms underlying the association between MetS and HF and to investigate how this association varies by sex and age, as well as the reasons behind these differences.

## 2. Metabolic Syndrome as a Risk Factor of Heart Failure

MetS and HF are closely linked through several interconnected pathophysiological mechanisms. The individual components of MetS—insulin resistance, visceral obesity, hypertension, and dyslipidemia—each contribute independently to an increased risk of HF.

Insulin resistance plays a central role in MetS, promoting hyperglycemia [20]. Hyperglycemia induces oxidative stress by disrupting cellular pathways, including the formation of advanced glycation end-products and elevated protein kinase C expression [21]. In turn, oxidative stress impairs cardiac mitochondrial function and leads to contractile dysfunction. It also activates fibrotic pathways, contributing to myocardial stiffness and fibrosis [22]. Additionally, insulin resistance increases lipolysis, resulting in elevated circulating levels of free fatty acids, which promote myocardial lipid accumulation through increased uptake. Insulin resistance is also linked to hyperactivation of the sympathetic nervous system and the renin–angiotensin–aldosterone system, which causes vasoconstriction, sodium retention, and elevated blood pressure. Collectively, these mechanisms associated with insulin resistance contribute to cardiac remodeling and hypertrophy, impairing cardiac function and ultimately leading to HF [11,12,13].

Visceral adiposity serves as a primary trigger, activating the pathways involved in MetS [23,24]. In the context of MetS, adipose tissue functions as an endocrine organ, producing biologically active molecules and inflammatory mediators. Adiponectin, a hormone produced by adipocytes, plays a key role that links MetS and HF. It enhances insulin sensitivity, and a high level of adiponectin is associated with improved metabolic regulation. In obesity, adiponectin is downregulated, contributing to insulin resistance, which in turn increases the risk of HF. Furthermore, adiponectin has anti-inflammatory properties, and in obesity, the secretion of pro-inflammatory cytokines such as tumor necrosis factor-alpha (TNF-α) and interleukin-6 (IL-6) increases, while adiponectin levels decrease. This imbalance creates a chronic inflammatory state, causing direct myocardial damage and promoting fibrosis. Obesity is also linked to hyperactivation of the sympathetic nervous system and the renin–angiotensin–aldosterone system, further contributing to HF development [11,13,20,25,26].

Hypertension, another key component of MetS, increases cardiac workload, leading to left ventricular hypertrophy (LVH). Over time, LVH can result in diastolic dysfunction and HF with preserved ejection fraction (HFpEF). Moreover, hypertension contributes to endothelial dysfunction, which reduces nitric oxide availability and promotes vasoconstriction, inflammation, and atherosclerosis, all of which impair coronary perfusion and cardiac function [11,14]. Moreover, the overproduction of angiotensin II by hypertrophic adipocytes leads to overactivation of the renin–angiotensin–aldosterone system in obesity, further exacerbating arterial vasoconstriction. The resulting overproduction of mineralocorticoid hormones contributes to myocardial tissue damage [27].

Dyslipidemia contributes to heart failure (HF) through multiple mechanisms. High-density lipoprotein (HDL) cholesterol plays a protective role by inhibiting cardiomyocyte hypertrophy through the suppression of angiotensin II type 1 receptor upregulation, a key factor in cardiac hypertrophy development [28], as well as through the inhibition of the Janus kinase/signal transducer and activator of transcription (JAK/STAT) pathway, which also promotes hypertrophy in cardiomyocytes [29,30,31]. Furthermore, HDL has anti-inflammatory [32,33,34,35], antioxidative [36,37,38], and endothelial-protective properties [39,40,41]. In the context of MetS, low HDL concentrations compromise these protective effects, increasing susceptibility to HF.

Additionally, metabolic abnormalities associated with MetS impair mitochondrial function, leading to decreased ATP production and increased reactive oxygen species generation. This mitochondrial dysfunction further contributes to cardiomyocyte dysfunction, promoting the progression of HF [11,14].

Several clinical studies have established MetS as an independent risk factor for HF. Our previous nationwide studies in individuals in their 40s and 50s demonstrated that the presence of MetS significantly increased the risk of HF [42,43]. After adjusting for known HF risk factors, such as body mass index (BMI), acute myocardial infarction, smoking, and family history of stroke, diabetes, or hypertension, the impact of MetS varied by age and sex, increasing the risk of HF by 1.7- to 2.4-fold overall [43]. A longitudinal study involving 2314 middle-aged men also identified MetS as a significant risk factor for HF, with a hazard ratio (HR) of 1.80 (95% confidence interval (CI), 1.11 to 2.91) [44]. A 20-year follow-up study of 1032 Finns, aged 65–74 years, found that MetS increases the incidence of HF, with an HR ranging from 1.37 to 1.87 after adjustment for confounding risk factors, depending on the MetS definition [45]. In another study of elderly individuals in their seventies, MetS was associated with an increased occurrence of HF hospital stay, with an HR of 1.49 (95% CI, 1.10 to 2.00) [46].

In summary, MetS is closely related to HF, being mechanistically interconnected with HF and established as a clinically significant risk factor for it.

## 3. The Association of Metabolic Syndrome and Heart Failure in Young Age

HF primarily affects the elderly population. While HF affects less than 2% of individuals aged 40–59, the prevalence rises to 6% in those aged 60–79 and reaches 14% in individuals over the age of 80 [47]. However, recent reports indicate a rising incidence of HF among younger adults. According to a 2017 report from Denmark, the annual incidence rates of HF (per 10,000 person–years) between 1995 and 2012 decreased in individuals over 55 years of age (164 versus 115 in individuals >74 years, 63 versus 35 in individuals 65–74 years, and 20 versus 17 in individuals 55–64 years), while it increased in those under 55 years of age (0.4 versus 0.7 in individuals 18–34 years, 1.3 versus 2.0 in individuals 35–44 years, and 5.0 versus 6.4 in individuals 45–54 years) [48]. A 2023 database study on HF patients in France also reported that between 2013 and 2018, the overall incidence of HF decreased in the general population but notably increased by approximately 0.041‰ among young adults [49]. Similarly, a study from Sweden investigating HF incidence from 1987 to 2006 showed that while HF incidence decreased in individuals over 45 years of age after the 1990s, it doubled in individuals under 45 years during the last five years of this study compared to the first five years [50].

The reasons for this increase in HF among younger adults are not clear. However, they are believed to be associated with the rising prevalence of risk factors for HF in this population, such as diabetes mellitus, morbid obesity, hypertension, cardiomyopathy [49,51], and myocarditis [52]. Notably, components of MetS, including diabetes, obesity, and hypertension, are key risk factors for both HF with reduced ejection fraction (HFrEF) and HFpEF [53], making it likely that the increasing prevalence of metabolic disorders among younger individuals is contributing to the rise in HF cases.

Several studies have shown that the impact of metabolic disorders on HF incidence is greater in younger adults compared to the elderly population. Tromp et al. [54] analyzed data from multiple observational cohort studies (the Framingham Heart Study (FHS) original and offspring cohorts, the Prevention of Renal and Vascular Endstage Disease (PREVEND) study, and the Multi-Ethnic Study of Atherosclerosis (MESA)) [55,56,57,58], integrating data from participants followed-up for 15 years. A total of 24,675 participants were divided into four age groups: young (<55 years), middle-aged (55–64 years), old (65–74 years), and elderly (≥75 years), to assess the influence of HF risk factors across different age groups. This study found that the impact of known metabolic HF risk factors was more pronounced in younger age groups compared to the elderly: HR for hypertension was 3.02 in young participants versus 1.43 in elderly participants; HR for diabetes was 3.86 in young participants versus 1.66 in elderly participants; and HR for obesity was 2.03 in young participants versus 1.73 in elderly participants.

Within young participants, the influence of metabolic factors on HF tended to be greater in younger age groups. Our research group analyzed the impact of MetS on HF in 1,958,284 individuals in their 40s [42] and 2,151,597 individuals in their 50s [43] in the Korean population. These two separate studies employed the same statistical method, i.e., the Cox proportional-hazards model, adjusting for other known risk factors. In both age groups, MetS was found to be an independent risk factor for HF. Within the same sex, the impact of MetS was greater in individuals in their 40s compared to those in their 50s; in men, HR was 2.0 for those in their 40s versus 1.7 for those in their 50s; and in women, it was 2.4 for those in their 40s versus 2.1 for those in their 50s. Even in cases of pre-MetS, where individuals had one or two components of MetS but did not meet the full diagnostic criteria, the impact was greater for those in their 40s than for those in their 50s (Table 1).

The reasons for the heightened risk of HF from metabolic disorders in younger individuals are not fully understood. In contrast to this study’s findings, mechanisms such as increased oxidative stress, DNA damage, impaired repair processes, and the gradual accumulation of myocardial fibrosis as individuals grow older may lower the threshold for HF development in the presence of metabolic disorders, making older adults more susceptible to its effects. One possible hypothesis is that the distinct components comprising MetS in younger versus older adults may contribute to these differences. Summer et al. [59] reported that MetS components vary with age, noting that younger adults with MetS often exhibit lower HDL cholesterol levels, less glucose intolerance, and lower hypertension prevalence. Specifically, the prevalence of low HDL cholesterol was 82.5% in those aged 30–39 and 75.5% in those aged 40–49 compared to 47.6% in adults over 70. Conversely, hypertension prevalence was lower in younger groups, with rates of 65.2% and 74.6% in the 30–39 and 40–49 age groups, respectively, compared to 94.7% in those over 70. While MetS is recognized as a considerable risk factor for HF, the relative impact of each MetS component on HF risk remains underexplored. If low HDL cholesterol exerts a stronger influence on HF development than other MetS components, this could explain the more significant impact of MetS on HF risk in younger versus older populations. Tromp et al. [54] suggested an additional view proposing that the less stringent management of metabolic conditions in younger adults may amplify their impact on HF development. In fact, some studies have reported the poorer management of hypertension in younger men compared to older men [60]. The development of metabolic disorders at a younger age may also be associated with poorer healthcare-seeking behavior, worse risk factor control, and lower socioeconomic status, all of which are independently linked to worse outcomes. However, considering that disease management in the elderly can also be suboptimal due to comorbidity or co-medication, this interpretation may not fully explain the observed phenomenon. Further investigation, including an analysis of the differential impact of each MetS component on HF in different age groups, is needed to better understand why MetS and metabolic disorders have a greater impact on HF development in younger populations.

## 4. Sex Difference in the Association of Metabolic Syndrome and Heart Failure

Multiple studies have reported that the metabolic abnormalities constituting MetS have differential impacts on the development of HF according to sex. These studies suggest that conditions such as diabetes mellitus, hypertension, and obesity, which arise from metabolic dysfunction, may confer a higher risk of HF in women than in men. For instance, Levy et al. [61], using data from the FHS original and offspring cohorts, analyzed 5143 participants and found that the presence of hypertension doubled the risk of HF in men, while it increased the risk by 3.4 times in women. Their multivariable analysis revealed that the population-attributable risk for HF due to hypertension was 39% in men and 59% in women. Regarding the relationship between obesity and HF, a study on 5881 FHS participants [62] showed that for each unit increase in BMI, the risk of HF increased by 4% in men and 7% in women. In this study, women who were overweight (25.0 ≤ BMI < 30.0) had a 68% higher risk of HF, and those who were obese (BMI ≥ 30.0) had double the risk compared to women with a normal BMI. In contrast, obese men had an 80% increased risk of heart failure compared to men with normal BMI, but being overweight was not identified as a significant risk factor for heart failure in men. A recent study [63] analyzing 22,681 individuals by HF subtype (HFpEF and HFrEF) found that obesity (BMI ≥ 30) increased the risk of HFpEF by 1.34 times in men and by 1.38 times in women. For HFrEF, obesity was a significant risk factor in men but not in women. The same study also examined the effect of central obesity (defined as a waist circumference ≥88 cm for women and ≥102 cm for men) and found that central obesity increased the risk of HFpEF more in women than in men. A study conducted by our research group on Korean individuals in their 40s [42] and 50s [43] also demonstrated sex differences in the impact of MetS on HF risk. In both age groups, women had a higher risk of developing HF than men. Compared to those with normal metabolic status, HF risk was 2.0 times higher in men vs. 2.4 times higher in women in their 40s and 1.7 times higher in men vs. 2.1 times higher in women in their 50s. Even in individuals with pre-MetS, HF risk was elevated, with women being more significantly affected than men (Table 2).

Although numerous studies have shown that metabolic disorders have a greater impact on HF development in women than in men, the reasons for this discrepancy remain unclear. Estrogen is believed to play a key role in sex-specific HF phenotypes, offering a protective effect against pressure overload [64,65] or in oxidative stress-induced injury [66,67,68], which contrasts with the greater susceptibility to HF in women with MetS than in men with MetS. One possible mechanism explaining this is sex differences in immune response. Research has shown that women generally exhibit higher pro-inflammatory cytokines, increased activation of inflammatory T cells, and elevated inflammatory markers such as C-reactive protein [69]. Furthermore, pro-inflammatory gene expression is more upregulated in the female myocardium [70]. These findings suggest that the inflammatory response triggered by metabolic dysregulation, including endothelial inflammation, may have a more pronounced role in HF development in women than in men. Another potential mechanism is the variation in nitric oxide bioavailability between sexes. Loyer et al. [71] discovered that after inducing pressure overload via thoracic aortic constriction, cardiac nitric oxide synthase expression was rapidly induced in male rats but delayed in female rats. This may indicate the sex difference in cardiac ability to adapt to hypertension. Additionally, studies showing that cardiometabolic risk factors exclusively increase left ventricular mass in women [72] and that reductions in natriuretic peptide levels in individuals with central obesity are observed only in women [73,74] indicate that MetS may affect cardiac muscles more significantly in women than in men. To better understand this difference, future studies could benefit from using biomarkers that indicate inflammation levels, nitric oxide availability, and hormone levels.

## 5. Conclusions

**Figure 1:**
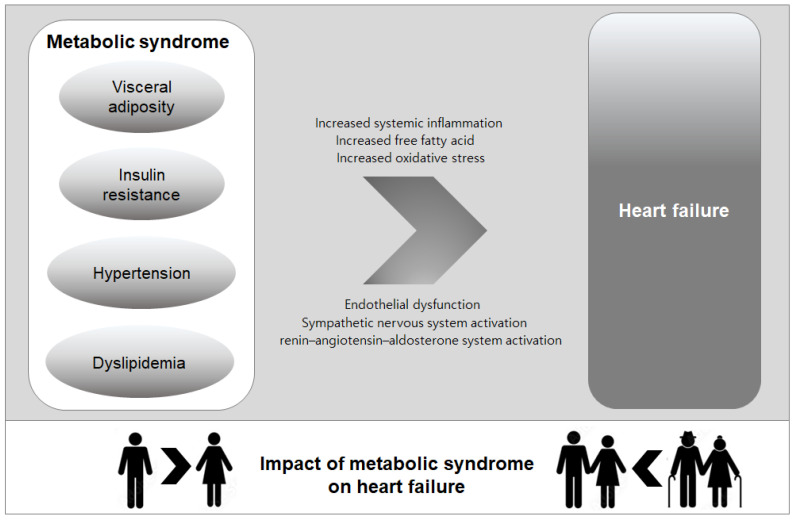
Pathophysiological link between metabolic syndrome and heart failure and the variation in the impact of metabolic syndrome on heart failure by sex and age.

This review highlights the significant associations between MetS and HF, with a particular focus on sex and age differences. The metabolic abnormalities constituting MetS—such as insulin resistance, hypertension, obesity, and dyslipidemia—have been shown to contribute to HF development through complex and multifaceted pathophysiological mechanisms. Importantly, these metabolic disruptions appear to have differential effects on HF risk based on sex and age, with women and younger individuals being particularly vulnerable (Figure 1).

Understanding the sex- and age-related differences in how MetS contributes to HF is crucial for developing more targeted prevention and treatment strategies. Tailored interventions based on individual metabolic profiles, with special consideration given to these demographic factors, may help reduce the burden of HF and improve long-term outcomes in patients with MetS. Future research should continue to explore the underlying mechanisms behind these variations and identify optimal management approaches that consider both sex and age in mitigating HF risk.

## Figures and Tables

**Table 1:** The impact of metabolic syndrome on heart failure development in Korean individuals in their 40s and 50s.

	Individuals in Their 40s		Individuals in Their 50s	
Metabolic Status	Men	Women	Men	Women
Normal	1	1	1	1
Pre-metabolic syndrome	1.607 (1.293–1.997)	1.893 (1.43–2.505)	1.508 (1.287–1.767)	1.395 (1.158–1.681)
Metabolic syndrome	1.968 (1.526–2.539)	2.398 (1.466–3.923)	1.711 (1.433–2.044)	2.144 (1.674–2.747)

Values are presented as hazard ratio (95% confidence interval).

**Table 2:** Sex difference in the impact of metabolic syndrome on the development of heart failure.

First Author, Year of Publication	Total Subjects, n	Population	Metabolic Disorder as a Risk Factor for HF	Main Findings (Risk for HF)
Levy et al. [46], 1996	5143	Original Framingham Heart Study and Framingham Offspring Study participants aged 40–89 years	Hypertension (SBP ≥ 140 mmHg or DBP ≥ 90 mmHg)	Population-attributable risk for CHF of 39% in men and 59% in women. Hazard for CHF of 2-fold in men and 3-fold in women.
Kenchaiah et al. [47], 2002	5881	Framingham Heart Study, mean age of 55 years	BMI (kg/m^2^), overweight (25.0 ≤ BMI < 30.0), obesity (BMI ≥ 30.0)	BMI (kg/m^2^) HR (95% CI): 1.04 (1.00–1.07) for men 1.07 (1.04–1.10) for women. Overweight HR (95% CI): 1.17 (0.86–1.61) for men and 1.68 (1.25–2.27) for women. Obesity HR (95% CI): 1.80 (1.22–2.64) for men and 2.17 (1.54–3.05) for women.
Savji et al. [48], 2019	22,681	Participants from 4 community-based cohorts, mean age of 60 years	BMI (kg/m^2^)	HFrEF HR (95% CI): 1.24(1.14–1.35) in men and 1.09 (0.96–1.24) in women. HFpEF HR (95% CI): 1.34 (1.18–1.52) in men and 1.38 (1.24–1.54) in women.
Kim et al. [29], 2022	2,151,597	Korea men and women, aged 50–60 years	MetS defined by NCEP ATP III	HR (95% CI): 1.711 (1.433–2.044) for men and 2.144 (1.674–2.747) for women
Kim et al. [28], 2024	1,958,284	Korea men and women, aged 40–50 years	MetS defined by NCEP ATP III	HR (95% CI): 1.968 (1.526–2.539) for men and 2.398 (1.466–3.923) for women

NCEP ATP III, National Cholesterol Education Program Adult Treatment Panel III; MetS, metabolic syndrome; CHF, congestive heart failure; SBP, systolic blood pressure; DBP, diastolic blood pressure; BMI, body mass index; HR, hazard ratio; CI, confidence interval; HFrEF, heart failure with reduced ejection fraction; HFpEF, heart failure with preserved ejection fraction.

## Data Availability

No new data were created or analyzed in this study. Data sharing is not applicable to this article.

## References

[1] Fahed G., Aoun L., Bou Zerdan M., Allam S., Bou Zerdan M., Bouferraa Y., Assi H.I. (2022). Metabolic Syndrome: Updates on Pathophysiology and Management in 2021. Int. J. Mol. Sci..

[2] Sattar N., Gaw A., Scherbakova O., Ford I., O’Reilly D.S., Haffner S.M., Isles C., Macfarlane P.W., Packard C.J., Cobbe S.M. (2003). Metabolic syndrome with and without C-reactive protein as a predictor of coronary heart disease and diabetes in the West of Scotland Coronary Prevention Study. Circulation.

[3] Isomaa B., Almgren P., Tuomi T., Forsen B., Lahti K., Nissen M., Taskinen M.R., Groop L. (2001). Cardiovascular morbidity and mortality associated with the metabolic syndrome. Diabetes Care.

[4] Lakka H.M., Laaksonen D.E., Lakka T.A., Niskanen L.K., Kumpusalo E., Tuomilehto J., Salonen J.T. (2002). The metabolic syndrome and total and cardiovascular disease mortality in middle-aged men. JAMA.

[5] Liang X., Or B., Tsoi M.F., Cheung C.L., Cheung B.M.Y. (2023). Prevalence of metabolic syndrome in the United States National Health and Nutrition Examination Survey 2011-18. Postgrad. Med. J..

[6] Bozkurt B., Coats A.J.S., Tsutsui H., Abdelhamid C.M., Adamopoulos S., Albert N., Anker S.D., Atherton J., Bohm M., Butler J. (2021). Universal definition and classification of heart failure: A report of the Heart Failure Society of America, Heart Failure Association of the European Society of Cardiology, Japanese Heart Failure Society and Writing Committee of the Universal Definition of Heart Failure: Endorsed by the Canadian Heart Failure Society, Heart Failure Association of India, Cardiac Society of Australia and New Zealand, and Chinese Heart Failure Association. Eur. J. Heart Fail..

[7] Savarese G., Becher P.M., Lund L.H., Seferovic P., Rosano G.M.C., Coats A.J.S. (2023). Global burden of heart failure: A comprehensive and updated review of epidemiology. Cardiovasc. Res..

[8] Braunwald E. (2013). Heart failure. JACC Heart Fail..

[9] Dunlay S.M., Shah N.D., Shi Q., Morlan B., VanHouten H., Long K.H., Roger V.L. (2011). Lifetime costs of medical care after heart failure diagnosis. Circ. Cardiovasc. Qual. Outcomes.

[10] Obi E.N., Swindle J.P., Turner S.J., Russo P.A., Altan A. (2016). Health Care Costs for Patients with Heart Failure Escalate Nearly 3-Fold in Final Months of Life. J. Manag. Care Spec. Pharm..

[11] Perrone-Filardi P., Paolillo S., Costanzo P., Savarese G., Trimarco B., Bonow R.O. (2015). The role of metabolic syndrome in heart failure. Eur. Heart J..

[12] Dei Cas A., Khan S.S., Butler J., Mentz R.J., Bonow R.O., Avogaro A., Tschoepe D., Doehner W., Greene S.J., Senni M. (2015). Impact of diabetes on epidemiology, treatment, and outcomes of patients with heart failure. JACC Heart Fail..

[13] Purwowiyoto S.L., Prawara A.S. (2021). Metabolic syndrome and heart failure: Mechanism and management. Med. Pharm. Rep..

[14] Gargiulo P., Marsico F., Renga F., Dell’Aversana S., Esposito I., Marciano C., Dellegrottaglie S., Perrone-Filardi P., Paolillo S. (2020). The metabolic syndrome in heart failure: Insights to specific mechanisms. Heart Fail. Rev..

[15] Miura Y., Fukumoto Y., Shiba N., Miura T., Shimada K., Iwama Y., Takagi A., Matsusaka H., Tsutsumi T., Yamada A. (2010). Prevalence and clinical implication of metabolic syndrome in chronic heart failure. Circ. J..

[16] Li C., Ford E.S., McGuire L.C., Mokdad A.H. (2007). Association of metabolic syndrome and insulin resistance with congestive heart failure: Findings from the Third National Health and Nutrition Examination Survey. J. Epidemiol. Community Health.

[17] Tamariz L., Hassan B., Palacio A., Arcement L., Horswell R., Hebert K. (2009). Metabolic syndrome increases mortality in heart failure. Clin. Cardiol..

[18] Perrone-Filardi P., Savarese G., Scarano M., Cavazzina R., Trimarco B., Minneci S., Maggioni A.P., Tavazzi L., Tognoni G., Marchioli R. (2015). Prognostic impact of metabolic syndrome in patients with chronic heart failure: Data from GISSI-HF trial. Int. J. Cardiol..

[19] Huang Z.M., Chen W.R., Su Q.W., Huang Z.W. (2021). Prognostic Impact of Metabolic Syndrome in Patients With Heart Failure: A Meta-Analysis of Observational Studies. Front. Cardiovasc. Med..

[20] Kostis J.B., Sanders M. (2005). The association of heart failure with insulin resistance and the development of type 2 diabetes. Am. J. Hypertens..

[21] Stratmann B., Tschoepe D. (2011). Heart in diabetes: Not only a macrovascular disease. Diabetes Care.

[22] Aimo A., Castiglione V., Borrelli C., Saccaro L.F., Franzini M., Masi S., Emdin M., Giannoni A. (2020). Oxidative stress and inflammation in the evolution of heart failure: From pathophysiology to therapeutic strategies. Eur. J. Prev. Cardiol..

[23] Pekgor S., Duran C., Berberoglu U., Eryilmaz M.A. (2019). The Role of Visceral Adiposity Index Levels in Predicting the Presence of Metabolic Syndrome and Insulin Resistance in Overweight and Obese Patients. Metab. Syndr. Relat. Disord..

[24] Matsuzawa Y., Funahashi T., Nakamura T. (2011). The concept of metabolic syndrome: Contribution of visceral fat accumulation and its molecular mechanism. J. Atheroscler. Thromb..

[25] Ouchi N., Parker J.L., Lugus J.J., Walsh K. (2011). Adipokines in inflammation and metabolic disease. Nat. Rev. Immunol..

[26] Oda E. (2008). The metabolic syndrome as a concept of adipose tissue disease. Hypertens. Res..

[27] Putnam K., Shoemaker R., Yiannikouris F., Cassis L.A. (2012). The renin-angiotensin system: A target of and contributor to dyslipidemias, altered glucose homeostasis, and hypertension of the metabolic syndrome. Am. J. Physiol. Heart Circ. Physiol..

[28] Lin L., Liu X., Xu J., Weng L., Ren J., Ge J., Zou Y. (2015). High-density lipoprotein inhibits mechanical stress-induced cardiomyocyte autophagy and cardiac hypertrophy through angiotensin II type 1 receptor-mediated PI3K/Akt pathway. J. Cell Mol. Med..

[29] Beckles D.L., Mascareno E., Siddiqui M.A. (2006). Inhibition of Jak2 phosphorylation attenuates pressure overload cardiac hypertrophy. Vascul Pharmacol..

[30] Pan J., Fukuda K., Saito M., Matsuzaki J., Kodama H., Sano M., Takahashi T., Kato T., Ogawa S. (1999). Mechanical stretch activates the JAK/STAT pathway in rat cardiomyocytes. Circ. Res..

[31] Jacoby J.J., Kalinowski A., Liu M.G., Zhang S.S., Gao Q., Chai G.X., Ji L., Iwamoto Y., Li E., Schneider M. (2003). Cardiomyocyte-restricted knockout of STAT3 results in higher sensitivity to inflammation, cardiac fibrosis, and heart failure with advanced age. Proc. Natl. Acad. Sci. USA.

[32] Carpintero R., Gruaz L., Brandt K.J., Scanu A., Faille D., Combes V., Grau G.E., Burger D. (2010). HDL interfere with the binding of T cell microparticles to human monocytes to inhibit pro-inflammatory cytokine production. PLoS ONE.

[33] Gomaraschi M., Basilico N., Sisto F., Taramelli D., Eligini S., Colli S., Sirtori C.R., Franceschini G., Calabresi L. (2005). High-density lipoproteins attenuate interleukin-6 production in endothelial cells exposed to pro-inflammatory stimuli. Biochim. Biophys. Acta.

[34] Wadham C., Albanese N., Roberts J., Wang L., Bagley C.J., Gamble J.R., Rye K.A., Barter P.J., Vadas M.A., Xia P. (2004). High-density lipoproteins neutralize C-reactive protein proinflammatory activity. Circulation.

[35] Murphy A.J., Woollard K.J., Suhartoyo A., Stirzaker R.A., Shaw J., Sviridov D., Chin-Dusting J.P. (2011). Neutrophil activation is attenuated by high-density lipoprotein and apolipoprotein A-I in in vitro and in vivo models of inflammation. Arterioscler. Thromb. Vasc. Biol..

[36] Bowry V.W., Stanley K.K., Stocker R. (1992). High density lipoprotein is the major carrier of lipid hydroperoxides in human blood plasma from fasting donors. Proc. Natl. Acad. Sci. USA.

[37] Navab M., Hama S.Y., Cooke C.J., Anantharamaiah G.M., Chaddha M., Jin L., Subbanagounder G., Faull K.F., Reddy S.T., Miller N.E. (2000). Normal high density lipoprotein inhibits three steps in the formation of mildly oxidized low density lipoprotein: Step 1. J. Lipid Res..

[38] Mackness M.I., Arrol S., Abbott C., Durrington P.N. (1993). Protection of low-density lipoprotein against oxidative modification by high-density lipoprotein associated paraoxonase. Atherosclerosis.

[39] Nofer J.R., Levkau B., Wolinska I., Junker R., Fobker M., von Eckardstein A., Seedorf U., Assmann G. (2001). Suppression of endothelial cell apoptosis by high density lipoproteins (HDL) and HDL-associated lysosphingolipids. J. Biol. Chem..

[40] Seetharam D., Mineo C., Gormley A.K., Gibson L.L., Vongpatanasin W., Chambliss K.L., Hahner L.D., Cummings M.L., Kitchens R.L., Marcel Y.L. (2006). High-density lipoprotein promotes endothelial cell migration and reendothelialization via scavenger receptor-B type I. Circ. Res..

[41] Cockerill G.W., Rye K.A., Gamble J.R., Vadas M.A., Barter P.J. (1995). High-density lipoproteins inhibit cytokine-induced expression of endothelial cell adhesion molecules. Arterioscler. Thromb. Vasc. Biol..

[42] Kim T.E., Kim D.Y., Kim H., Sung J., Kim D.K., Lee M.S., Han S.W., Kim H.J., Ki H.K., Kim S.H. (2024). The Impact of Metabolic Syndrome on Heart Failure in Young Korean Population: A Nationwide Study. Metabolites.

[43] Kim T.E., Kim H., Sung J., Kim D.K., Lee M.S., Han S.W., Kim H.J., Kim S.H., Ryu K.H. (2022). The association between metabolic syndrome and heart failure in middle-aged male and female: Korean population-based study of 2 million individuals. Epidemiol. Health.

[44] Ingelsson E., Arnlov J., Lind L., Sundstrom J. (2006). Metabolic syndrome and risk for heart failure in middle-aged men. Heart.

[45] Wang J., Sarnola K., Ruotsalainen S., Moilanen L., Lepisto P., Laakso M., Kuusisto J. (2010). The metabolic syndrome predicts incident congestive heart failure: A 20-year follow-up study of elderly Finns. Atherosclerosis.

[46] Butler J., Rodondi N., Zhu Y., Figaro K., Fazio S., Vaughan D.E., Satterfield S., Newman A.B., Goodpaster B., Bauer D.C. (2006). Metabolic syndrome and the risk of cardiovascular disease in older adults. J. Am. Coll. Cardiol..

[47] Benjamin E.J., Virani S.S., Callaway C.W., Chamberlain A.M., Chang A.R., Cheng S., Chiuve S.E., Cushman M., Delling F.N., Deo R. (2018). Heart Disease and Stroke Statistics-2018 Update: A Report From the American Heart Association. Circulation.

[48] Christiansen M.N., Kober L., Weeke P., Vasan R.S., Jeppesen J.L., Smith J.G., Gislason G.H., Torp-Pedersen C., Andersson C. (2017). Age-Specific Trends in Incidence, Mortality, and Comorbidities of Heart Failure in Denmark, 1995 to 2012. Circulation.

[49] Lecoeur E., Domeng O., Fayol A., Jannot A.S., Hulot J.S. (2023). Epidemiology of heart failure in young adults: A French nationwide cohort study. Eur. Heart J..

[50] Barasa A., Schaufelberger M., Lappas G., Swedberg K., Dellborg M., Rosengren A. (2014). Heart failure in young adults: 20-year trends in hospitalization, aetiology, and case fatality in Sweden. Eur. Heart J..

[51] Park M.J., Paul Mulye T., Adams S.H., Brindis C.D., Irwin C.E. (2006). The health status of young adults in the United States. J. Adolesc. Health.

[52] Heymans S., Eriksson U., Lehtonen J., Cooper L.T. (2016). The Quest for New Approaches in Myocarditis and Inflammatory Cardiomyopathy. J. Am. Coll. Cardiol..

[53] Golla M.S.G., Hajouli S., Ludhwani D. (2024). Heart Failure and Ejection Fraction. StatPearls.

[54] Tromp J., Paniagua S.M.A., Lau E.S., Allen N.B., Blaha M.J., Gansevoort R.T., Hillege H.L., Lee D.E., Levy D., Vasan R.S. (2021). Age dependent associations of risk factors with heart failure: Pooled population based cohort study. BMJ.

[55] Dawber T.R., Kannel W.B., Lyell L.P. (1963). An approach to longitudinal studies in a community: The Framingham Study. Ann. N. Y Acad. Sci..

[56] Kannel W.B., Feinleib M., McNamara P.M., Garrison R.J., Castelli W.P. (1979). An investigation of coronary heart disease in families. The Framingham offspring study. Am. J. Epidemiol..

[57] Bild D.E., Bluemke D.A., Burke G.L., Detrano R., Diez Roux A.V., Folsom A.R., Greenland P., Jacob D.R., Kronmal R., Liu K. (2002). Multi-Ethnic Study of Atherosclerosis: Objectives and design. Am. J. Epidemiol..

[58] Brouwers F.P., de Boer R.A., van der Harst P., Voors A.A., Gansevoort R.T., Bakker S.J., Hillege H.L., van Veldhuisen D.J., van Gilst W.H. (2013). Incidence and epidemiology of new onset heart failure with preserved vs. reduced ejection fraction in a community-based cohort: 11-year follow-up of PREVEND. Eur. Heart J..

[59] Sumner A.D., Sardi G.L., Reed J.F. (2012). Components of the metabolic syndrome differ between young and old adults in the US population. J. Clin. Hypertens..

[60] Daugherty S.L., Masoudi F.A., Ellis J.L., Ho P.M., Schmittdiel J.A., Tavel H.M., Selby J.V., O’Connor P.J., Margolis K.L., Magid D.J. (2011). Age-dependent gender differences in hypertension management. J. Hypertens..

[61] Levy D., Larson M.G., Vasan R.S., Kannel W.B., Ho K.K. (1996). The progression from hypertension to congestive heart failure. JAMA.

[62] Kenchaiah S., Evans J.C., Levy D., Wilson P.W., Benjamin E.J., Larson M.G., Kannel W.B., Vasan R.S. (2002). Obesity and the risk of heart failure. N. Engl. J. Med..

[63] Savji N., Meijers W.C., Bartz T.M., Bhambhani V., Cushman M., Nayor M., Kizer J.R., Sarma A., Blaha M.J., Gansevoort R.T. (2018). The Association of Obesity and Cardiometabolic Traits With Incident HFpEF and HFrEF. JACC Heart Fail..

[64] Zhai P., Eurell T.E., Cooke P.S., Lubahn D.B., Gross D.R. (2000). Myocardial ischemia-reperfusion injury in estrogen receptor-alpha knockout and wild-type mice. Am. J. Physiol. Heart Circ. Physiol..

[65] Zhai P., Eurell T.E., Cotthaus R., Jeffery E.H., Bahr J.M., Gross D.R. (2000). Effect of estrogen on global myocardial ischemia-reperfusion injury in female rats. Am. J. Physiol. Heart Circ. Physiol..

[66] Miller V.M., Duckles S.P. (2008). Vascular actions of estrogens: Functional implications. Pharmacol. Rev..

[67] Stirone C., Duckles S.P., Krause D.N., Procaccio V. (2005). Estrogen increases mitochondrial efficiency and reduces oxidative stress in cerebral blood vessels. Mol. Pharmacol..

[68] Essop M.F., Chan W.Y., Taegtmeyer H. (2007). Metabolic gene switching in the murine female heart parallels enhanced mitochondrial respiratory function in response to oxidative stress. FEBS J..

[69] Klein S.L., Flanagan K.L. (2016). Sex differences in immune responses. Nat. Rev. Immunol..

[70] InanlooRahatloo K., Liang G., Vo D., Ebert A., Nguyen I., Nguyen P.K. (2017). Sex-based differences in myocardial gene expression in recently deceased organ donors with no prior cardiovascular disease. PLoS ONE.

[71] Loyer X., Oliviero P., Damy T., Robidel E., Marotte F., Heymes C., Samuel J.L. (2007). Effects of sex differences on constitutive nitric oxide synthase expression and activity in response to pressure overload in rats. Am. J. Physiol. Heart Circ. Physiol..

[72] Lieb W., Xanthakis V., Sullivan L.M., Aragam J., Pencina M.J., Larson M.G., Benjamin E.J., Vasan R.S. (2009). Longitudinal tracking of left ventricular mass over the adult life course: Clinical correlates of short- and long-term change in the framingham offspring study. Circulation.

[73] Suthahar N., Meijers W.C., Ho J.E., Gansevoort R.T., Voors A.A., van der Meer P., Bakker S.J.L., Heymans S., van Empel V., Schroen B. (2018). Sex-specific associations of obesity and N-terminal pro-B-type natriuretic peptide levels in the general population. Eur. J. Heart Fail..

[74] De Boer R.A., Nayor M., deFilippi C.R., Enserro D., Bhambhani V., Kizer J.R., Blaha M.J., Brouwers F.P., Cushman M., Lima J.A.C. (2018). Association of Cardiovascular Biomarkers With Incident Heart Failure With Preserved and Reduced Ejection Fraction. JAMA Cardiol..

